# Hazelnut (*Corylus avellana* L.) Shells Extract: Phenolic Composition, Antioxidant Effect and Cytotoxic Activity on Human Cancer Cell Lines

**DOI:** 10.3390/ijms18020392

**Published:** 2017-02-13

**Authors:** Tiziana Esposito, Francesca Sansone, Silvia Franceschelli, Pasquale Del Gaudio, Patrizia Picerno, Rita Patrizia Aquino, Teresa Mencherini

**Affiliations:** 1 Department of Pharmacy, University of Salerno, Via Giovanni Paolo II, 132, I-84084 Fisciano (SA), Italy; tesposito@unisa.it (T.E.); fsansone@unisa.it (F.S.); sfranceschelli@unisa.it (S.F.); pdelgaudio@unisa.it (P.D.G.); ppicerno@unisa.it (P.P.); aquinorp@unisa.it (R.P.A.); 2 Ph.D. Program in Drug Discovery and Development, University of Salerno, Via Giovanni Paolo II 132, I-84084 Fisciano (SA), Italy

**Keywords:** hazelnut by-product, neolignans, diaryleptanoid, DPPH radical, caspase-3, PARP-1

## Abstract

Hazelnut shells, a by-product of the kernel industry processing, are reported to contain high amount of polyphenols. However, studies on the chemical composition and potential effects on human health are lacking. A methanol hazelnut shells extract was prepared and dried. Our investigation allowed the isolation and characterization of different classes of phenolic compounds, including neolignans, and a diarylheptanoid, which contribute to a high total polyphenol content (193.8 ± 3.6 mg of gallic acid equivalents (GAE)/g of extract). Neolignans, lawsonicin and cedrusin, a cyclic diarylheptanoid, carpinontriol B, and two phenol derivatives, C-veratroylglycol, and β-hydroxypropiovanillone, were the main components of the extract (0.71%–2.93%, *w*/*w*). The biological assays suggested that the extract could be useful as a functional ingredient in food technology and pharmaceutical industry showing an in vitro scavenging activity against the radical 1,1-diphenyl-2-picrylhydrazyl radical (DPPH) (EC_50_ = 31.7 μg/mL with respect to α-tocopherol EC_50_ = 10.1 μg/mL), and an inhibitory effect on the growth of human cancer cell lines A375, SK-Mel-28 and HeLa (IC_50_ = 584, 459, and 526 μg/mL, respectively). The expression of cleaved forms of caspase-3 and poly(ADP-ribose) polymerase-1 (PARP-1) suggested that the extract induced apoptosis through caspase-3 activation in both human malignant melanoma (SK-Mel-28) and human cervical cancer (HeLa) cell lines. The cytotoxic activity relies on the presence of the neolignans (balanophonin), and phenol derivatives (gallic acid), showing a pro-apoptotic effect on the tested cell lines, and the neolignan, cedrusin, with a cytotoxic effect on A375 and HeLa cells.

## 1. Introduction

Hazelnut (*Corylus avellana* L., Betulaceae family) is one of the most cultivated and marketed nuts in the world. Italy is the second largest hazelnut-producing area (about 105,000 t/year), behind Turkey (about 600,000 t/year) [1]. About 10% of the world crop production is sold as in-shell product consumed fresh or roasted, and the remaining 90% as shelled hazelnuts and used as an ingredient in food (bakery, confectionary industry, and chocolate) processing industries [2,3]. During the kernel harvesting and industrial processing, a large amount of by-products, including green leafy cover, shell and skin, is obtained. Their disposal represents both an economic problem for the producers and a serious environmental problem due to the combustion of the crop residues [4,5,6]. The ligno-cellulose shells, obtained after cracking the kernel, account for the majority of this waste, and they are used as a heat source, for mulching, and furfural production in dye manufacturing [7]. The reported antioxidant potential of both hazelnut kernel and shell extracts might be related to the presence of phenolic acids and tannins [8,9,10,11]. Polyphenols have received great attention for their human health benefits due to antioxidant properties [8,11,12,13]. Intake of foods or vegetable products rich in polyphenols is generally recognized as useful for the prevention and treatment of cancer, and cardiovascular, inflammatory, microbial, and age-related diseases [14]. In particular, the chemopreventive efficacy of these natural antioxidants has been demonstrated against several human cancer cell lines [15]. Therefore, recovery and upgrading of hazelnut shells seems to be consistent with the growing demand for ingredients that have beneficial effects on human health. Nevertheless, the information about the chemical profile of hazelnut shells is limited to the identification of free and bound phenolic compounds, such as flavonoid glycosides and aromatic acids, in hazelnuts cultivated in Poland [16]. Therefore, the aim of the present study was to define the chemical composition and biological activities of the methanol extract from hazelnut shells (HSE). The research led to the isolation and characterization by Nuclear Magnetic Resonance (NMR) and Elettrspray Mass Spectrometry (ESI-MS) of four neolignans with a dihydro[*b*]benzofuran skeleton, seven phenolic derivatives, and a cyclic diarylheptanoid. The in vitro free radical scavenging activity of HSE and isolated compounds was determined by DPPH test. The antiproliferative activity of HSE and its major components against human melanoma (primary and metastatic, A375, and SK-Mel-28, respectively) and cervical cancer (HeLa) cell lines was evaluated by MTT bioassay. The potential pro-apoptotic mechanism of action, as well as the involvement of caspase-3 and its major substrate PARP-1 in the apoptotic process, was investigated.

## 2. Results and Discussion

### 2.1. Extract Preparation, Chemical Composition, and Quantitative Analysis

**Figure 1:**
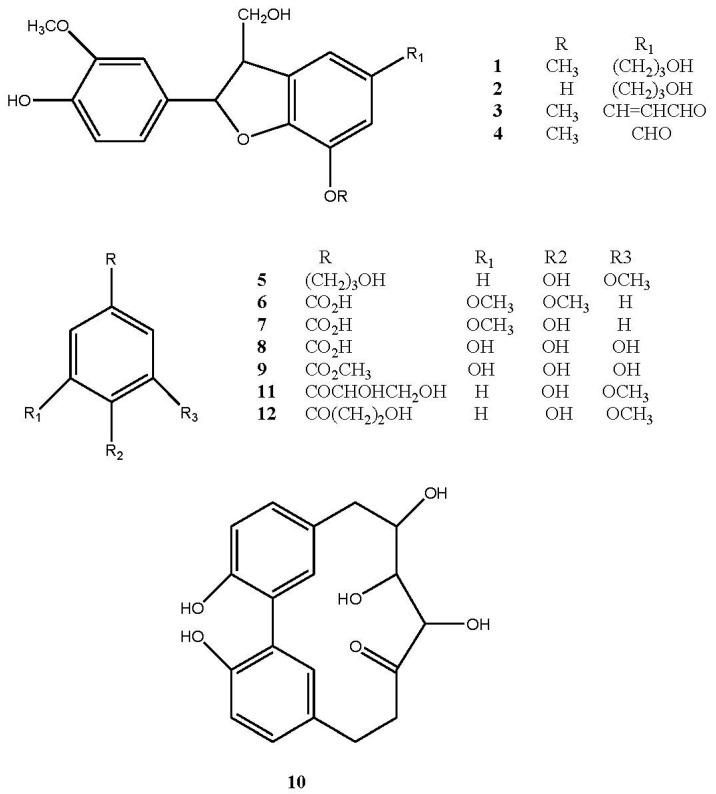
Structures of compounds (**1**–**12**) isolated from hazelnut shells extract (HSE).

In order to investigate the chemical profile and biological activities of hazelnut shells, a methanol extract (HSE) from powdered and defatted shells was prepared. The extraction yield, after maceration (3 times × 24 h) at room temperature of shells, was about 2.08%. This result is comparable to that reported by Shahidi et al. (2007) [9] and Contini et al. (2008) [8] using aqueous ethanol, methanol, or acetone as solvent systems and hot-reflux extractor (80 °C) or a long maceration at room temperature as extraction procedures. A portion of HSE (1.5 g) was subjected to chromatography by Sephadex LH-20 and RP-HPLC to obtain twelve major constituents belonging to different phenolic subclasses. The structures of the isolated compounds (Figure 1) were established by their NMR and MS data in comparison to those found in the literature. They include four dihydro[*b*]benzofuran-type neolignans (**1**–**4**), lawsonicin (**1**) [17], cedrusin (**2**) [18], balanophonin (**3**) [19], and ficusal (**4**) [20]; seven phenolic derivatives, dihydroconiferyl alcohol (**5**) [21], veratric acid (**6**) [22], vanillic acid (**7**) [17], gallic acid (**8**), methyl gallate (**9**) [23], C-veratroylglycol (**11**) [24], and β-hydroxypropiovanillone (**12**) [25]; and a cyclic diarylheptanoid, carpinontriol B (**10**) [26]. Vanillic and gallic acids (**7**–**8**) have been previously identified in hazelnut kernel and shells [16], while the presence in hazelnut of compounds **1**–**6** and **9**–**12** was revealed for the first time.

**Figure 2:**
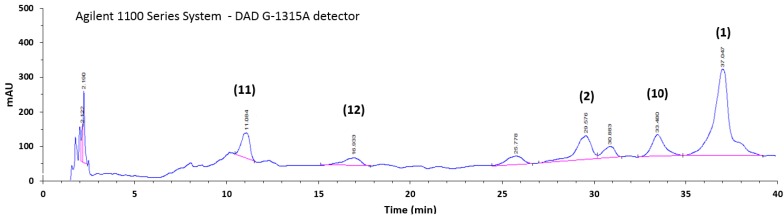
HPLC-DAD fingerprint (230 nm) of hazelnut shells extract (HSE). The peak numbers in this figure correspond to compounds in Figure 1.

The major components of HSE, neolignans (**1**) and (**2**), cyclic diarylheptanoid (**10**), and phenols (**11**) and (**12**), were selected as markers of the extract and their quantitative analysis was performed by High-Performance Liquid Chromatography with Diode-Array Detection (HPLC-DAD) using the isolated compounds as the standards for calibration curves. The HPLC fingerprint is reported in Figure 2. Lawsonicin (**1**), cedrusin (**2**), carpinontriol B (**10**), C-veratroylglycol (**11**), and β-hydroxypropiovanillone (**12**) were found to be 1.98%, 1.79%, 1.41%, 2.93%, and 0.71%, *w*/*w* of the extract, respectively. Other isolated compounds (**3**–**9**) were not quantified.

### 2.2. Free Radical Scavenging Activity

The well known antioxidant activity of phenolic compounds is generally thought to be due to redox properties, which can play an important role in neutralizing free radicals, quenching singlet and triplet oxygen, or decomposing peroxides [27]. Considering their occurrence in HSE, the free-radical scavenging activity of the extract was verified by DPPH test. This method evaluates the ability of a sample to scavenge the chromogen long-lived DPPH free radical [28]. Results (Table 1) showed that the extract possessed a significant and concentration-dependent free radical scavenging (EC_50_ = 31.7 μg/mL) which may be correlated to its high polyphenol content, evaluated by Folin–Ciocalteu method, and expressed as gallic acid equivalent (193.8 mg GAE/g of the extract). Moreover, the free-radical scavenging activity of all isolated compounds was also evaluated, with the aim to identify the compounds responsible for HSE activity. As previously reported [23], gallic acid (**8**) and methyl gallate (**9**), water-soluble polyphenols, were very effective in quenching free-radicals, exhibiting an EC_50_ of 1.2 and 1.4 μg/mL, respectively, 10-fold higher than α-tocopherol (EC_50_ = 10.1 μg/mL) used as positive control (Table 1). Neolignans, lawsonicin (**1**), cedrusin (**2**), and balanophonin (**3**), phenolic acid derivatives, vanillic (**6**) and veratric (**7**) acids, and cyclic diarylheptanoid, carpinotriol B (**10**) had EC_50_ values ranging from 42.7 to 89.2 μg/mL (Table 1). Only ficusal (**4**) and dihydroconiferyl alcohol (**5**) were about 10-fold less active than α-tocopherol (Table 1). Results were in agreement with the observation that the structure of polyphenols is the key determinant of their antioxidant activity [29]. The strong effect of phenolic acids such as gallic acid (**8**) and methyl gallate (**9**) is due to three free hydroxyl groups at position 3, 4 and 5 on the aromatic ring [30]. The loss of a hydroxyl group and/or the presence of one or more methoxy groups on the aromatic ring reduced drastically the activity as observed for veratric (**7**) and vanillic (**6**) acids (Table 1), respectively. Moreover, in the series of di-ortho phenolic derivatives, β-hydroxypropiovanillone (**12**) was more active than C-veratroylglycol (**11**) and dihydroconiferyl alcohol (**5**) (Table 1), probably due to modification in the side chain. Considering the structures of neolignans **1**–**4**, the free-radical scavenging activity was as follows **2** > **3** > **1** > **4**, suggesting that the effect could be related to the 3-phenylpropan-1-ol unit and free hydroxyl group at position C-3′ [31]. The presence of a methoxy group at C-3′ (lawsonicin, **1** and balanophonin, **3**) decreases the efficacy; and the activity disappeared in ficusal (**4**) which shows the loss of the side chain and presence of an aldheide function at R_1_. In conclusion, the significant free radical scavenging activity of the hazelnut shells extract could be ascribed to the additive and synergistic effect of its phenols, which may exert, in combination, a better antiradical effect than individual compound [32].

### 2.3. Cytotoxic Activity of Hazelnut Shells Extract (HSE) and Isolated Compounds

The treatment of melanoma and cervical cancer with conventional chemotherapy, surgery, and radiation, alone or in combination, is rather unsatisfactory [15,33]. Therefore, the research on functional foods fortified and enriched with natural potential chemopreventive additives, dietary supplements, and nutraceuticals able to decrease the incidence of these cancers, is raising a great interest. The cytotoxic activities of gallic acid and neolignans with a dihydro[*b*]benzofuran against several cancer cell lines have been reported [14,16,31,34]. In the present study, the activity of hazelnut extract and its constituents in inhibiting cell proliferation was evaluated by MTT assay against human melanoma (primary A375, metastatic SK-Mel-28), and cervical cancer (HeLa) cell lines. The total extract (HSE) exhibited a significant (*p* < 0.05) and concentration-dependent inhibitory effect on the tumor cell lines growth (IC_50_ 459–584 μg/mL, Table 2). Balanophonin (**3**), and gallic acid (**8**) were cytotoxic on all cell lines with IC_50_ values ranging from 142 to 200 μM (Table 2). The neolignan cedrusin (**2**) was found active in A375 and HeLa cells (IC_50_ = 130 and 141 μM, respectively) for the first time. On the contrary, other neolignans, lawsonicin (**1**) and ficusal (**4**), phenol derivatives, dihydroconyferyl alcohol (**5**), veratric acid (**6**), vanillic acid (**7**), C-veratroylglycol (**11**), and β-hydroxypropiovanillone (**12**), and cyclic diarylheptanoid, carpinontriol B (**10**) were not cytotoxic up to 1000 μM (Table 2). Results indicated that the effect of HSE on cancer cell growth might be due to a synergy of action of the neolignans, cedrusin (**2**) and balanophonin (**3**), and gallic acid (**8**). However, it cannot be excluded that not isolated or interfering constituents may contribute to the extract activity.

Gallic acid has been shown to induce apoptosis in cancer cells and it has been recognized as a chemopreventive agent [33,35,36]. However, there is no study in the literature supporting the possible mechanism of action of neolignans, cedrusin (**2**), and balanophonin (**3**), in the induction of human cancer cell death. Therefore, the potential apoptotic effect of the extract, HSE, and the most cytotoxic neolignans (**2**) and (**3**), and gallic acid (**8**) was investigated evaluating the presence of hypodiploid nuclei in the cells by flow-cytometric analysis, after incubating with the extract (100–500 μg/mL) or compounds (each 100–500 μM) for 24 h [17].

**Figure 3:**
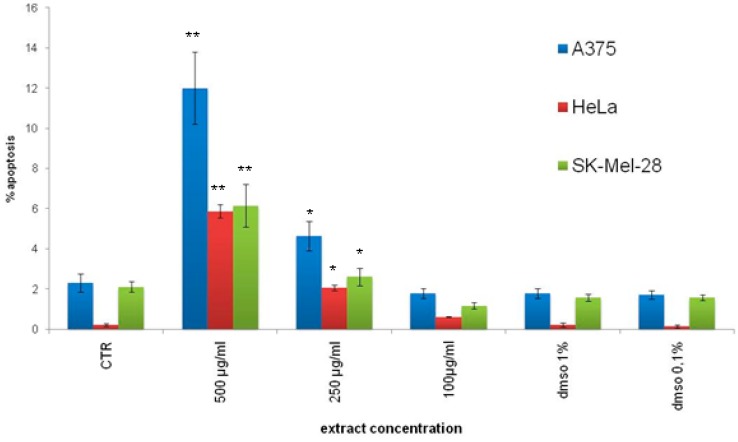
Effects of hazelnut shells extract (HSE) on apoptosis of A375, SK-Mel-28 and HeLa cells. Analysis of percentage of nuclei in apoptosis was performed with propidium iodide staining. Cancer cells were incubated with different concentrations of hazelnut shells extract (HSE) (100–500 μg/mL) for 24 h. Cells were then collected, and the percentage of hypodiploid nuclei was analyzed by flow cytometry (* *p* < 0.05, ** *p* < 0.01 vs. control cells). All results are shown as mean ± standard deviation of three experiments performed in triplicate. Statistical comparison between groups were made using ANOVA followed by the Bonferroni parametric test. Differences were considered significant if *p* < 0.05.

Figure 3 shows that the extract induced apoptosis in all treated cancer cells increasing in a dose-dependent manner the percentage of hypodiploid nuclei. Notably, this effect was significant (*p* < 0.05) from 250 μg/mL and was more evident in A375 cells compared to SK-Mel-28 and HeLa cells. Moreover, compounds (**2**), (**3**) and (**8**) exhibited a pro-apoptotic effect (data not shown).

One of the most common signaling cascades involved in apoptosis is the activation of caspases, a family of cysteinyl-aspartate proteases, usually present as inactive zymogen forms. Caspases cleave several proteins, during the execution phase of apoptosis, and among them, PARP-1 (poly(ADP-ribose) polymerase-1), a nuclear enzyme involved in DNA repair, DNA replication, and modulation of chromatin structure [37]. In response to genotoxic stress, PARP-1 is cleaved by caspase-3 and -7 into a ~25 kDa N-terminal fragment, containing the DNA binding domain (DBD), and a ~85 kDa C-terminal fragment that retains basal enzymatic activity PARP-1, recognizes DNA strand interruptions, and can complex with RNA inhibiting transcription. Through these processes, PARP-1 cleavage may help cells to commit to the apoptotic pathway [38,39].

**Figure 4:**
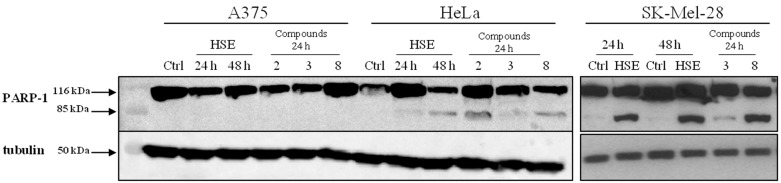
PARP-1 expression in A375, HeLa and SK-Mel-28 cell lines after 24 and 48 h of treatment. Hazelnut shells extract (HSE) and compounds (after 24 h) induce PARP-1 cleavage in HeLa and SK-Mel-28, but not in A375 cell lines.

In order to investigate the mechanism of apoptosis induction by both the extract (HSE) and compounds in A375, SK-Mel-28 and HeLa cancer cells, the expressed levels of caspase-3 and PARP-1 cleavage were further analyzed by Western blotting analysis. Results indicated that HSE, balanophonin (**3**), and gallic acid (**8**) induced PARP-1 cleavage after 24–48 h of treatment (Figure 4) in human cervical cancer (HeLa) and human malignant metastatic melanoma (SK-Mel-28) cell lines. Therefore, hazelnut extract and compounds-induced apoptosis is mediated by caspase-3 activation in the above cancer cells. Conversely, no activation of PARP-1 in human malignant melanoma (A375) cells (Figure 4) suggested that the pro-apoptotic mechanism of extract and compounds must be further investigated.

## 3. Materials and Methods

### 3.1. Chemicals and Reagents

Analytical grade *n*-hexane, chloroform, *n*-butanol, and methanol employed for extraction and isolation procedures, methanol deuterated, Folin–Ciocalteu phenol reagent, 1,1-diphenyl-2-picrylhydrazyl radical (DPPH), α-tocopherol, and HPLC-grade methanol were purchased from Sigma-Aldrich (Milan, Lombardia, Italy). HPLC-grade water (18 mΩ) was prepared by a Milli-Q_50_ purification system (Millipore Corp., Bedford, MA, USA). Water and MeOH employed for the electrospray ionization ESI-MS analysis were of HPLC supergradient quality (Romil Ltd., Cambridge, UK). Human malignant melanoma (A375, and SK-Mel-28), human cervical cancer (HeLa), all reagents, and supplements for cell cultures were obtained from Gibco Life Technology Corp. (ThermoFischer Scientific, Milan, Italy). Sodium citrate, Triton X-100 and propidium iodide (PI) were purchased from (Sigma-Aldrich, St. Louis, MO, USA). PARP-1 (F-2) antibody was acquired from Santa Cruz Biotechnology, Inc. (Heidelberg, Germany).

### 3.2. General Experimental Procedures

A Bruker DRX-600 NMR spectrometer (Bruker Italia, Milano, Italia), operating at 599.19 MHz for ^1^H and 150.858 MHz for ^13^C, using the TopSpin 3.2 software package (Bruker Italia, Milano, Italy), was used for NMR experiments in CD_3_OD. Chemical shifts are expressed in δ (parts per million) referring to the solvent peaks δ_H_ 3.31 and δ_C_ 49.05 for CD_3_OD, with coupling constants, *J*, in Hertz. Conventional pulse sequences were used for ^1^H-^1^H DQF-COSY (Double Quantum Filter-Correlation Spectroscopy) ^1^H-^13^C HSQC (Heteronuclear Single Quantum Coherence), and HMBC (Heteronuclear Multiple Bond Correlation) experiments [27]. ESI-MS experiments were performed with a Finnigan LC-Q Deca spectrometer (Thermoquest, San Jose, CA, USA), equipped with Xcalibur 3.1 software (Thermoquest, San Jose, CA, USA). Chromatography was performed on Sephadex LH-20 (Pharmacia, Uppsala, Sweden). Thin-layer chromatography (TLC) analysis was performed with Macherey−Nagel precoated silica gel 60 F_254_ plates (Delchimica, Naples, Italy), and the spray reagent cerium sulfate (saturated solution in dilute H_2_SO_4_) and UV (254 and 366 nm) were used for the spot visualization. Preparative HPLC separations were conducted on a Waters 590 series pumping system, equipped with a Waters R401 refractive index detector and a Rheodyne injector (100 μL loop), using μ-Bondapak C_18_ (300 × 7.8 mm i.d., 10 μm, Waters) or Luna C_8_ (250 × 10.0 mm i.d., 10 μm, Phenomenex, Torrance, CA, USA) as column. An Agilent 1100 series system (Agilent Technologies, Waldbronn, Germany), equipped with a Model G-1312 pump, a Rheodyne Model G-1322A loop (20 μL), and a DAD G-1315A detector was used for the HPLC quantitative analysis using a Nucleodur 100-5 C_18_ column (150 × 4.6 mm, 5 μm, Machery-Nagel). Peaks area were calculated with an Agilent Integrator (Agilent Technologies, Waldbronn, Germany).

### 3.3. Materials

Hazelnut shells were provided from a local company, Hazelnuts South Italy Manufacturing S.r.l. (Baiano, Avellino, Italy). They represented the waste of industrial processing carried out on two Italian varieties (90% Mortarella and 10% Lunga San Giovanni) at roasting temperature of 240 °C for 30 min. The shells were ground in a mortar grinder (RM 100, Retsch, Bergamo, Italy) for 5 min. The shells (1000 g) were sequentially defatted with *n*-hexane and chloroform, and extracted at room temperature (3 times × 1.6 L for 24 h) with methanol. The organic solvent was removed under vacuum at 40 °C in a rotary evaporator (Rotavapor R-200, Buchi Italia s.r.l, Cornaredo, Italy), to give 20.8 g of residue (HSE). The extraction yield, gravimetrically determined (balance Denver Instruments-PK-201; 15/30 °C), and expressed as the weight percentage of the dry matter compared to the total amount of the initial material, was 2.08%, *w*/*w*.

### 3.4. Isolation Procedure of Compounds **1**–**12**

A portion of the dried HSE (1.5 g) was fractionated using a Sephadex LH-20 column (1 m × 5 cm) with MeOH as eluent at flow rate 0.5 mL/min. Fractions of 8 mL each were collected, and combined into six major groups (**I**–**VI**) based on their TLC spots (Si-gel, *n*-BuOH–acetic acid–H_2_O (60:15:25, *v*/*v*/*v*), CHCl_3_–MeOH–H_2_O (7:3:0.3, *v*/*v*/*v*). Fractions **I**, **III** and **V**–**VI** were purified by RP-HPLC on a C_8_ column (flow rate 2.0 mL/min) with the elution solvent MeOH/H_2_O 4:6 *v*/*v*. Fraction **I** (545.0 mg) yielded compounds **5** (6.3 mg, *t_R_* = 15 min), and **2** (33.4 mg, *t_R_* = 26 min), while fraction **III** (99.0 mg) afforded compounds **6** (9.2 mg, *t_R_* = 20 min), and **7** (1.3 mg, *t_R_* = 32 min). Fraction **V** (38.8 mg) consisted of compounds **8** (1.8 mg, *t_R_* = 8 min), and **9** (0.2 mg, *t_R_* = 14 min). Fraction **VI** (105.2 mg) gave compound **10** (2.8 mg, *t_R_* = 42 min). Fraction **II** (114.8 mg) was separated by RP-HPLC on a C_8_ column (flow rate 1.5 mL/min) using as solvent system MeOH/H_2_O 4:6 *v*/*v* to afford compounds **11** (5.4 mg, *t_R_* = 8 min), **12** (3.2 mg , *t_R_* = 14 min), **2** (2.3 mg, *t_R_* = 24 min), and **1** (2.5 mg, *t_R_* = 54 min). Finally, fraction **IV** (53.8 mg) was purified by RP-HPLC using MeOH/H_2_O 5:5 *v*/*v* on a C_18_ column (flow rate 2.0 mL/min) to obtain compounds **4** (1.7 mg, *t_R_* = 14 min), and **3** (2.0 mg, *t_R_* = 19 min).

### 3.5. Spectroscopic Data

Lawsonicin (**1**): NMR and optical rotation data were consistent with those previously reported [17]. ESI-MS (positive mode), *m*/*z* 361.4 [M + H]^+^. Cedrusin (**2**): NMR and optical rotation data were consistent with previously reported [18]*.* ESI-MS (positive mode), *m*/*z* 347.3 [M + H]^+^. Balanophonin (**3**): NMR data were consisted with previously reported [19]. ESI-MS (positive mode), *m*/*z* 357.3 [M + H]^+^. Ficusal (**4**): NMR and optical rotation data were consistent with those previously reported [20]. ESI-MS (positive mode), *m*/*z* 331.1 [M + H]^+^. Dihydroconyferyl alcohol (**5**): NMR data were consistent with previously reported [21]. ESI-MS (positive mode), *m*/*z* 183.2 [M + H]^+^. Veratric acid (**6**): NMR data were consistent with previously reported [22]. ESI-MS (negative mode), *m*/*z* 181.1 [M − H]^−^. Vanillic acid (**7**). NMR data were consisted with previously reported [17]. ESI-MS (negative mode), *m*/*z* 167.1 [M − H]^−^. Gallic acid (**8**) and methyl gallate (**9**): NMR data were in agreement with those previously reported [23]. ESI-MS (negative mode), *m*/*z* 169.1 [M − H]^−^ and 183.1 [M – H]^−^, respectively. Carpinontriol B (**10**): NMR and optical rotation data were consistent with those previously reported [26]. ESI-MS (positive mode), *m*/*z* 344.1 [M + H]^+^. C-veratroylglycol (**11**): NMR data were in agreement with those previously reported [24]. ESI-MS (positive mode), *m*/*z* 213.3 [M + H]^+^. β*-*hydroxypropiovanillone (**12**): NMR data were in agreement with those previously reported [25]. ESI-MS (positive mode), *m*/*z* 197.0 [M + H]^+^.

### 3.6. Quantitative Determination of Total Phenol Content

Total phenolic content (TPC) of hazelnut shells extract (HSE) was determined using the Folin–Ciocalteu colorimetric method [4]. TPC was expressed as gallic acid equivalents (GAE) mg/g of dried HSE (means ± standard deviation of three determinations).

### 3.7. Quantitative HPLC Analysis of HSE

Quantitative HPLC was carried out using as eluent system H_2_O (solvent A) and MeOH (solvent B). The solvent gradient was as follows: 0→3 min, 5% B; 3→7 min, 5%→30% B; 7→17 min, 30% B; 17→35 min, 30%→50% B, 40→50 min 100% B. Elution was performed with a flow rate of 0.8 mL/min, injection volume of 20 μL, and DAD detector set at 230 nm. Analysis was carried out in triplicate. Lawsonicin (**1**), C-veratroylglycol (**11**), and cedrusin (**2**) (isolated from HSE and characterized by NMR, and MS data) were used to prepare three solutions at different concentration levels in the range 0.25–1.00 mg/mL for compounds **1** and **11**, and 0.25–2.00 mg/mL for **2**. The peak associated with each compound was identified by comparison of the retention times, and confirmed by co-injection of HSE with isolated compounds. Peak areas of isolated compounds lawsonicin (**1**), cedrusin (**2**), carpinontriol B (**10**), C-veratroylglycol (**11**), and β-hydroxypropiovanillone (**12**) (at each concentration) were plotted against the corresponding standard concentrations (mg/mL) using linear regression to generate standard curves (regression equation *y* = 30885.7*x* − 1704.1, *r* = 0.9989 for **1**; *y* = 16723.0*x* − 1348.2, *r* = 0.9997 for **2**; *y* = 11109*x* + 213.7, *r* = 0.9980 for **10**; *y* = 8761.8*x* + 104.0, *r* = 1.0000 for **11**, *y* = 14914*x* − 460.22, *r* = 0.9984 for **12**, where *y* is the peak area and *x* the concentration). HSE was dissolved in MeOH at 10 mg/mL, and analyzed under the same chromatographic conditions.

### 3.8. Antioxidant Activity

The radical scavenging activities of HSE and compounds **1**–**12** were assayed using stable 1,1-diphenyl-2-picrylhydrazyl radical (DPPH), according to our procedures previously reported [4]. Briefly, 1.5 mL of DPPH solution (25 mg/mL in methanol, prepared daily) was added to 0.375 mL of various concentrations, in MeOH solution, of each sample under investigation (ranged from 12 to 100 μg/mL). The mixtures were kept in the dark for 10 min at room temperature and the decrease in absorbance was measured at 517 nm against a blank consisting of an equal volume of methanol. α-Tocopherol was used as positive control. The DPPH concentration in the reaction medium was calculated from a calibration curve (range = 5–36 μg/mL) analyzed by linear regression (*y* = 0.0228*x* − 0.0350, *R*^2^ = 0.9999), and EC_50_ (mean effective scavenging concentration) was determined as the concentration (in micrograms per milliliter) of sample necessary to decrease the initial DPPH concentration by 50%. All tests were performed in triplicate.

### 3.9. Cell Cultures

Human malignant melanoma (A375), and Human cervical cancer (HeLa) cell lines were grown at 37 °C in Dulbecco’s modified Eagle’s medium containing high glucose supplemented with 10% fetal calf serum, and 100 units/mL each of penicillin and streptomycin, and 2 mmol/L glutamine. Human melanoma (SK-Mel-28) cell line was grown at 37 °C in minimum essential medium (MEM) supplemented with 10% fetal calf serum and 100 units/mL each of penicillin and streptomycin. At the onset of each experiment, cells were placed in fresh medium and then cultured in the presence of different concentrations of HSE or its constituents. The experiments were repeated three times.

### 3.10. Cell Viability Assay

To perform the assay, the cells were grown in 96-well plates, in numbers of 7000 per well and after 24 h were treated with increasing concentrations of HSE from 10 μg/mL to 1 mg/mL and with isolated compounds from 10 nM to 500 μM, in triplicate for a given time (24 and 48 h). At the end of treatment, the plates were centrifuged at 1200 rpm for 5 minutes, the medium was aspirated and added 100 μL of 1 mg/mL MTT (3-[4,5-dimetiltiazol-2,5-diphenyl-2H-tetrazolium bromide]) to each well and the plates were kept at 37 °C for the time necessary to the formation of salt formazan (1–3 h depending on cell type). The solution was then removed from each well, and the formazan crystal within the cells were dissolved with 100 μL of DMSO. Absorption at 550 nm for each well was assessed by a Multiskan Spectrum Thermo Electron Corporation Reader. IC_50_ values were calculated from cell viability dose–response curves and defined as the concentration resulting in 50% inhibition of cell survival compared to untreated cells.

### 3.11. Flow Cytometry Analysis

Apoptosis was analyzed by propidium iodide incorporation in permeabilized cells and flow cytometry [17,40]. After 24 h of culture in 24-wells plates, cancer cells (5 × 10^4^) were treated with HSE or compounds at different doses, and re-cultured for 24 or 48 h. The apoptosis analysis was carried out in permeabilized cells labelled with propidium iodide (PI) by incubation at 4 °C for 30 min with a solution containing 0.1% sodium citrate, 0.1% Triton X-100 and 50 mg/mL PI. Subsequently, the cancer cells were analyzed by flow cytometry by a FACSCalibur flow cytometer (Becton Dickinson, North Ryde, NSW, Australia). Each experiment was repeated three times.

### 3.12. Western Blotting Analysis

Cells were lysed in modified RIPA buffer (Tris-HCl pH 7.4 10 mM, NaCl 150 mM, EDTA 1 mM, NP40 1% Na-deoxycholic 0.1%, PMSF 1 mM, protease inhibitor cocktail). Equal amounts of proteins were separated by 10%–12% SDS-PAGE and blotted on ECl Hybond nitro-cellulose membranes (GE Healthcare, Buckinghamshire, UK). Blots were blocked in PBS containing 10% non-fat dry milk and 0.1% Tween-20 and incubated overnight with optimal dilutions of PARP-1 (F-2) antibody for detection of full-lenght and the C-terminal cleavage product (95 kDa) of PARP-1. Anti-mouse IgG HRP conjugated were used as secondary antibody, bands were visualized by autoradiography of ECL reaction (Pierce, Thermo Scientific, Rockford, IL, USA), and anti α-tubulin antibody were used as control for equal amounts of proteins loaded on the gel.

### 3.13. Statistical Analysis

All results are shown as mean ± standard deviation of three experiments performed in triplicate. Statistical comparison between groups were made using ANOVA followed by the Bonferroni parametric test. Differences were considered significant when *p* < 0.05.

## 4. Conclusions

Few chemical and biological studies on hazelnut shells, a waste product of industrial food processing, have been reported in the literature until now. The present research contributes to further understand the composition and bioactivity of hazelnut shells. Neolignans, dihydro[*b*]benzofuran-type (lawsonicin, cedrusin, balanophonin, and ficusal), phenolic derivatives (dihydroconyferyl alcohol, veratric, vanillic and gallic acids, methyl gallate, C-veratroylglycol, and β-hydroxypropiovanillone), and a cyclic diarylheptanoid (carpinontriol B) are the main constituents of the hazelnut methanol extract and these phytochemicals, with the exception of vanillic and gallic acids, are found in hazelnut for the first time. The extract exhibited an in vitro significant free-radical scavenging activity that was mainly due to gallic acid and its methyl ester. Both compounds were proven to be potential free-radical scavengers in the methanol extracts. The hazelnut extract, some neolignans, cedrusin (**2**) and balanophonin (**3**) and gallic acid (**8**) are able to inhibit the growth of human cancer cells (primary melanoma, A375, metastatic melanoma, SK-Mel-28, and cervical cancer, HeLa) inducing apoptosis mediated by caspase-3 activation and PARP-1 cleavage. Thus, extracts from hazelnut shells might be useful as health-promoting ingredients potentially expandable in functional foods, nutraceuticals or dietary supplements.

## Figures and Tables

**Table 1:** Total Phenolic Content and free-radical scavenging activity of hazelnut shells extract (HSE) and compounds **1**–**12**.

Extract and Compounds	Phenol Content (mg/g Extract) ^a^	EC_50_^b^ (μg/mL)
HSE	193.8 ± 3.6 ^c^	31.7 ± 1.4 ^c^
**1**		74.3 ± 3.8
**2**		42.7 ± 2.5
**3**		59.2 ± 2.9
**4**		160.0 ± 4.5
**5**		118.7 ± 3.5
**6**		55.4 ± 1.2
**7**		58.6 ± 3.5
**8**		1.2 ± 0.2
**9**		1.9 ± 0.8
**10**		78.2 ± 2.1
**11**		89.2 ± 3.2
**12**		54.6 ± 2.8
α-Tocopherol ^d^		10.1 ± 1.3

^a^ Gallic acid equivalent; ^b^ EC_50_ ± standard deviation (data from three experiments in triplicate); ^c^ Mean ± SD of three determination by the Folin–Ciocalteu method; ^d^ Positive control of the DPPH assay.

**Table 2:** Effect of hazelnut shells extract (HSE) and its compounds on human cancer cell lines.

Extract or Compound	Cell Line		
	A375 ^a^ (IC_50_) ^b^	SK-Mel-28 (IC_50_)	HeLa (IC_50_)
HSE	584.0 ± 9.0 ^c^	459.0 ± 8.3	526.0 ± 8.9
1	NA ^d^	NA	NA
2	130.0 ± 4.2	NA	141.0 ± 3.8
3	142.0 ± 3.6	150.0 ± 4.1	143.0 ± 4.4
4	NA	NA	NA
5	NA	NA	NA
6	NA	NA	NA
7	NA	NA	NA
8	170.0 ± 3.2	150.0 ± 4.0	200.0 ± 3.3
10	NA	NA	NA
11	NA	NA	NA
12	NA	NA	NA

^a^ A375 and SK-Mel-28, melanoma cells; HeLa, cervical cancer cells; ^b^ IC_50_, required concentration of hazelnut shells extract or pure compound to inhibit cell proliferation by 50% expressed as μg/mL for extract and μM for compounds; ^c^ IC_50_ ± standard deviation (data from three experiments in triplicate); and ^d^ Not active (IC_50_ > 1000 μM).
